# Extracellular Neuroleukin Enhances Neuroleukin Secretion From Astrocytes and Promotes Axonal Growth *in vitro* and *in vivo*

**DOI:** 10.3389/fphar.2018.01228

**Published:** 2018-10-30

**Authors:** Yoshitaka Tanie, Norio Tanabe, Tomoharu Kuboyama, Chihiro Tohda

**Affiliations:** Division of Neuromedical Science, Department of Bioscience, Institute of Natural Medicine, University of Toyama, Toyama, Japan

**Keywords:** astrocytes, neuroleukin, axonal growth, 78 kDa glucose regulated protein, spinal cord injury

## Abstract

Under pathological conditions in the central nervous system (CNS), including spinal cord injury, astrocytes show detrimental effects against neurons. It is also known that astrocytes sometimes exert beneficial effects, such as neuroprotection and secretion of axonal growth factors. If beneficial effects of astrocytes after injury could be induced, dysfunction of the injured CNS may improve. However, a way of promoting beneficial functions in astrocytes has not been elucidated. In the current study, we focused on neuroleukin (NLK), which is known to have axonal growth activities in neurons. Although NLK is secreted from astrocytes, the function of NLK in astrocytes is poorly understood. We aimed to clarify the mechanism of NLK secretion in astrocytes and the functional significance of secreted NLK from astrocytes. Stimulation of cultured astrocytes with recombinant NLK significantly elevated the secretion of NLK from astrocytes. Furthermore, astrocyte conditioned medium treated with NLK increased axonal density in cultured cortical neurons. Recombinant NLK itself directly increased axonal density in cultured neurons. These results indicated that NLK secreted from astrocytes acted as an axonal growth factor and that secretion was stimulated by extracellular NLK. To elucidate a direct binding molecule of NLK on astrocytes, drug affinity responsive target stability (DARTS) analysis was performed. A 78 kDa glucose regulated protein (GRP78) was identified as a receptor for NLK, which was related to the secretion of NLK from astrocytes. When NLK was injected into the lesion site of spinal cord injured mice, axonal density in the injured region was significantly increased and hindlimb motor function improved. These results suggested that NLK-GRP78 signalling was important for the beneficial effects of astrocytes. This study strengthens the potential of astrocytes for use as therapeutic targets in CNS traumatic injury.

## Introduction

Astrocytes are multifunctional cells that have important roles in homeostasis of neural networks in the central nervous system (CNS). Under pathological conditions in the CNS, such as traumatic injury of the brain and spinal cord, astrocytes exert both detrimental and beneficial effects on neural networks (Karimi-Abdolrezaee and Billakanti, 2012; Karve et al., 2016), including neurotoxic effects after injury (Liddelow et al., 2017). In addition, CNS traumatic injury induces the activation of astrocytes that form glial scars surrounding the lesion site. In the glial scar, chondroitin sulphate proteoglycans (CSPGs) are deposited and inhibit axonal regeneration (Silver and Miller, 2004). In contrast, the formation of a glial scar is also considered to play beneficial roles for protecting healthy tissues from neurotoxic environments (Okada et al., 2006; Shinozaki et al., 2017). Furthermore, astrocytes are reported to express and secrete axonal growth factors such as brain-derived neurotrophic factor, nerve growth factor, and periostin in the injured spinal cord (Dougherty et al., 2000; Krenz and Weaver, 2000; Shih et al., 2014). We previously showed that in spinal cord injured (SCI) mice, astrocytes also promote axonal growth and functional recovery via secretion of vimentin, which is an axonal growth factor (Teshigawara et al., 2013; Shigyo et al., 2015; Shigyo and Tohda, 2016). If the beneficial effects of astrocytes could be increased after injury, CNS function might be improved. However, the mechanism to increase beneficial astrocyte activity is poorly understood.

Neuroleukin (NLK), also called autocrine motility factor (AMF), has been identified as a cytokine secreted from tumour cells (Liotta et al., 1986). Secreted NLK shows autocrine activity to promote cell motility in tumour cells (Silletti et al., 1991; Watanabe et al., 1991). In cultured chondrocytes, treatment with recombinant NLK enhances production and secretion of NLK, suggesting that NLK secretion is regulated by a positive feedback mechanism (Tian et al., 2015). In addition, previous studies reported that NLK shows pro-survival effects in cultured sensory neurons (Gurney et al., 1986) and axonal growth effects in the co-culture of neural stem cells and sertoli cells (Deng et al., 2014). Although NLK is also secreted from cultured astrocytes (Dowell et al., 2009), the signalling involved in the promotion of NLK secretion and the roles of secreted NLK have not been elucidated. We postulated that NLK promotes NLK secretion in astrocytes through its autocrine activity and that secreted NLK induces axonal growth in neurons. In the current study, we aimed to clarify the mechanism of NLK secretion in astrocytes and the functional significance of NLK secreted from astrocytes.

## Materials and Methods

All experiments were performed in accordance with the Guidelines for the Care and Use of Laboratory Animals of the University of Toyama. The Committee for Animal Care and Use at the Sugitani Campus of the University of Toyama approved the study protocols. The approval number for the animal experiments was A2016INM-3. All efforts were made to minimise the number of animals used.

### Primary Cultures

Primary cultured cerebral cortical neuronal cells were prepared from ddY mice (Japan SLC, Hamamatsu, Japan) at embryonic day 14 (E14) as described previously (Tohda et al., 2012). Eight-well chamber slides coated with 5 μg/ml poly-D-lysine (PDL; Sigma-Aldrich, St. Louis, MD, United States) were used for cultures. The cells were cultured on PDL-coated slides with neurobasal medium (Life Technologies, Carlsbad, CA, United States) containing 12% horse serum (Life Technologies), 0.6% glucose, 2 mM L-glutamine (HS medium) at 37°C in a humidified incubator with 10% CO_2_. Four hours later, the medium was replaced with fresh neurobasal medium containing 2% B-27 supplement (Life Technologies) without horse serum (B27 medium).

Mouse spinal cord astrocytes were cultured as described previously (McCarthy and de Vellis, 1980; Marek et al., 2008) with modifications. Briefly, mouse spinal cord cells (E14, ddY mice) were cultured for 8–11 days and then shaken for 15–18 h in T-25 flasks (Falcon) with Dulbecco’s modified Eagle’s media (DMEM)/F12 (1:1) medium (Life Technologies) containing 10% foetal bovine serum at 37°C in a humidified incubator with 10% CO_2_. Microglia and oligodendrocyte precursors were detached by shaking. The isolated astrocytes were seeded onto 8-well chamber slides or 48-well plates (Thermo Fisher Scientific, Waltham, MA, United States) at a density of 1.3–2.7 × 10^5^ cells/cm^2^. At 7 days *in vitro*, the cells were fixed with 4% paraformaldehyde and immunostained with a rabbit anti-glial fibrillary acidic protein (GFAP) polyclonal antibody (1:1000; Cat. No. AB5804, Millipore, Burlington, MA, United States) as an astrocyte marker. Alexa Fluor 594-conjugated goat anti-rabbit IgG (1:400; Cat. No. A-11012, Life Technologies) was used as a secondary antibody. Nuclei were counterstained with 1 μg/ml 4′,6-diamidino-2-phenylindole (DAPI; Enzo Life Sciences, Farmingdale, NY, United States). Fluorescent images were captured using a Cell Observer Z1 fluorescent microscope (Carl Zeiss, Oberkochen, Germany). GFAP-positive cells and GFAP-non-positive cells were counted using MetaMorph version 7.8 (Molecular Devices, Sunnyvale, CA, United States). The purity of GFAP-positive astrocytes was quantified with ratio of GFAP-positive cells to total cells. The purity of GFAP-positive astrocytes was approximately 75% (data not shown).

### Quantification of NLK in Astrocyte Lysate and Conditioned Medium

Previous study showed the dosages of recombinant NLK in the range of ng/ml for cultured chondrocytes (Tian et al., 2015). In this study, therefore, cultured spinal cord astrocytes were treated with 10, 100, 300, or 500 ng/ml recombinant NLK (Cat. No. ATGP0348, ATGen, Seongnam, South Korea) or vehicle (sterile distilled water). Six days post treatment, the cells were washed with neurobasal medium containing 0.6% glucose, 2 mM L-glutamine (supplement-free medium) and then incubated with fresh supplement-free medium. The cells were cultured for 24 h to generate astrocyte conditioned medium (ACM). The cells were treated with the following neutralising antibodies: an anti-AMF receptor antibody (1 μg/ml, Cat. No. NBP2-15734, Novus Biologicals, Littleton, CO, United States) (Lucarelli et al., 2015) and an anti-78 kDa glucose regulated protein (GRP78) antibody (2 μg/ml, Cat. No. cs-1050, Santa Cruz Biotechnology, Dallas, TX, United States) (Kelber et al., 2009). As a negative control, normal rabbit immunoglobulin (IgG; 1 μg/ml, Cat. No. sc-2027, Santa Cruz Biotechnology) or normal goat IgG (2 μg/ml, Cat. No. sc-2028, Santa Cruz Biotechnology) was used. In our preliminary experiments, when neutralising antibodies were treated to cultured astrocytes with concentrations used in previous reports (Kelber et al., 2009; Lucarelli et al., 2015), those concentrations of the antibody showed cell toxicity. Therefore, the concentration of the neutralising antibodies used were based on the maximum concentration with no cell toxicity (data not shown). After 15 min of treatment with the neutralising antibody, 500 ng/ml recombinant NLK or vehicle was administered to the cells and incubated for 24 h. After washing, fresh supplement-free medium was added and the cells cultured for 24 h to prepare ACM. The ACM was collected, filtered using a 0.22 μm filter (Millipore), and concentrated with Amicon Ultra-0.5 10K Centrifugal Filter Unit (Millipore) by centrifugation at 14,000 × *g*, 50 min, 4°C. The protein concentration in the ACM was measured using a NanoOrange Protein Quantitation Kit (Thermo Fisher Scientific).

For the preparation of cell lysate, the cells were washed with phosphate-buffered saline (PBS) and then incubated with mammalian protein extraction reagent (M-PER) lysis buffer (Thermo Fisher Scientific) containing a protease and phosphate inhibitor cocktail (Thermo Fisher Scientific) for 20 min on ice. After incubation, the cell solution was centrifuged (14,000 × *g*, 10 min, 4°C) to remove cell debris, and the supernatants were used as cell lysates. The cell lysate protein concentration was measured using a Pierce 660 nm Protein Assay Kit (Thermo Fisher Scientific). Lysates were mixed with NuPAGE lithium dodecyl sulphate (LDS) sample buffer (Life Technologies) containing 5% 2-mercaptoethanol (Wako, Osaka, Japan) at 75°C for 5 min and loaded onto an 8% sodium dodecyl sulphate-polyacrylamide gel (SDS–PAGE). After electrophoresis, proteins in the gel were transferred to a nitrocellulose membrane (Bio-Rad, Berkeley, CA, United States) and blocked with 0.1% Tween 20 in tris buffered saline (T-TBS) containing 5% skim milk (Wako) at room temperature. Subsequently, the membrane was gently washed with T-TBS and incubated with a mouse monoclonal anti-NLK antibody (1:1000; Cat. No. ab66340, Abcam, Cambridge, United Kingdom) in Can Get Signal solution 1 (Toyobo, Osaka, Japan) overnight at 4°C. After washing with T-TBS, the membrane was incubated with a horse-radish peroxidase (HRP)-conjugated secondary antibody against mouse IgG (1:2000; Cat. No. sc-2005, Santa Cruz) in Can Get Signal solution 2 (Toyobo) for 2 h at room temperature. After washing, the membrane was reacted with electron chemiluminescence (ECL) Prime Western Blotting Detection Reagent (GE Healthcare, Buckinghamshire, United Kingdom) and chemiluminescence on the membrane was detected using an ImageQuant LAS 4000 system (GE Healthcare). Antibodies on the membrane were then stripped with western blotting stripping solution (Nacalai Tesque, Kyoto, Japan) and the membrane incubated with an anti-β-actin rabbit polyclonal antibody (1:1000; Cat. No. 4970, Cell Signaling Technology, MA, United States) in Can Get Signal solution 1 overnight at 4°C. After washing with T-TBS, the membrane was incubated with an HRP-conjugated secondary antibody against rabbit IgG (1:2000) in Can Get Signal solution 2 for 2 h at room temperature. Chemiluminescence on the membrane was detected as described above. The signal intensities were quantified using a CS analyser (ATTO, Tokyo, Japan).

### Axonal Growth Assay

In case of neuron culture on PDL, cortical neurons were cultured for 1 day *in vitro* and then treated with 10, 50, or 100 ng/ml NLK or with ACM for 5 days. In case of neuron culture on CSPG, the cells were cultured in CSPG (2 μg/ml Aggrecan; Sigma-Aldrich) coated wells for 1 day *in vitro* and then treated with 10 or 100 ng/ml NLK for 4 days. Four or five days post treatment, the cells were fixed with 4% paraformaldehyde and immunostained with a mouse anti-phosphorylated neurofilament-H (pNF-H) monoclonal antibody (1:300; Cat. No. SMI-35R, Covance, Emeryville, CA, United States) as an axonal marker and a rabbit anti-microtubule-associated protein 2 (MAP2) polyclonal antibody (1:2000; Cat. No. ab32454, Abcam) as a neuronal marker. Alexa Fluor 594-conjugated goat anti-mouse IgG (1:400; Cat. No. A-11005, Life Technologies) and Alexa Fluor 488-conjugated goat anti-rabbit IgG (1:400; Cat. No. A-11008, Life Technologies), respectively, were used as secondary antibodies. Nuclei were counterstained with 1 μg/ml 4′,6-diamidino-2-phenylindole (DAPI; Enzo Life Science). Fluorescent images were captured using a Cell Observer Z1 fluorescent microscope (Carl Zeiss) at a photo size of 432.49 μm × 322.81 μm. The lengths of pNF-H-positive axons were measured using MetaMorph version 7.8 (Molecular Devices). The sum of the axon lengths was divided by the number of MAP2-positive neurons in each photo to calculate the mean axonal length.

### Identification of Putative Direct Binding Protein With Extracellular NLK in Cultured Astrocytes by Drug Affinity Responsive Target Stability (DARTS) Analysis

Drug affinity responsive target stability analysis was performed as described previously (Tohda et al., 2012). Cell lysate of cultured astrocytes containing 10 μg protein was added to 0.1, 1, or 10 μg/ml recombinant NLK or vehicle and incubated for 30 min at room temperature. Thereafter, the mixture was proteolysed with thermolysin (Wako) in reaction buffer containing 50 mM Tris–HCl, pH 8.0; 50 mM NaCl; 10 mM CaCl_2_ for 30 min at 37°C (thermolysin:protein, 1:10 μg). At the end of the reaction period, 0.5 M ethylenediaminetetraacetic acid (pH 8.0) was added to each sample at a 1:10 ratio on ice to stop proteolysis. Samples were incubated with NuPAGE LDS sample buffer (Life Technologies, Carlsbad, CA, United States) and 5% 2-mercaptoethanol at 75°C for 5 min. The samples were loaded onto 8% polyacrylamide gels and electrophoresed. The gels were incubated in fix solution (40% ethanol, 10% acetic acid in ultrapure water) at room temperature overnight. The proteins in the gels were silver stained for visualisation using a SilverQuest Kit (Invitrogen, Carlsbad, CA, United States). A protein band (indicated by the red arrowhead in Figure 4A) was thinner in the sample treated with 10 μg/ml NLK compared to that of the sample treated with vehicle. The band was excised from the gel, digested with trypsin, and then analysed by mass spectrometry using a Nano liquid chromatography-tandem mass spectrometry (LC-MS/MS) system (Japan Bio Services, Saitama, Japan). A candidate protein from the electrophoresis band was identified as GRP78 using UniProt and MASCOT databases and the spectrum data (sequence coverage: 30%, score: 630). To confirm whether the candidate protein was GRP78, western blotting was performed using samples after proteolysis in the DARTS analysis. Western blotting was performed as described above using an anti-GRP78 antibody (1:1000; Santa Cruz) for labelling. Membrane-associated lysate was prepared from cultured astrocytes at 6 days *in vitro* culturing using a membrane protein extraction reagent (Mem-PER) Plus Membrane Protein Extraction Kit (Thermo Fisher Scientific) following manufacturer protocol.

### Microinjection of NLK to SCI Mice

All mice were housed with *ad libitum* access to food and water and kept in a constant environment (22 ± 2°C, 50 ± 5% humidity, 12 h light cycle starting at 07:00). Eight-week-old female ddY mice (SLC, Japan) were used for SCI experiments. The mice were anaesthetised with trichloroacetaldehyde monohydrate (500 mg/kg, i.p.). After laminectomy, contusion injury was established by dropping a 6.5-g weight from a height of 3 cm onto the exposed spinal cord at the level of T11–T12 using a stereotaxic instrument (NARISHIGE, Tokyo, Japan) as described previously (Shigyo and Tohda, 2016). The microinjection of recombinant NLK (1 mg/ml) or vehicle (20 mM Tris–HCl, 1 mM dithiothreitol, 10% glycerol) into the lesion site was performed within 15 min after injury at two sites (lateral ± 1.0 mm, depth 0.7 mm from the lesion centre). Each solution (1 μl/site) was injected via glass capillaries (Probeta) at a rate of 0.25 μl/min using a KDS-210 syringe pump (KD Scientific, Holliston, MA, United States). The inner diameter of the tip of the glass capillaries was processed to 40 μm using a PC-10 puller (NARISHIGE) and an MF-900 microforge (NARISHIGE). Basso mouse scale (BMS) (Basso et al., 2006) and Toyama mouse score (TMS) (Shigyo et al., 2014) were used to evaluate hindlimb motor function of the SCI mice in an open field (black colour, 50.0 cm × 42.5 cm × 15.0 cm) under 500-lux illumination. The behavioural evaluation was started 1 day post injury and performed once a day for 20 days.

### Immunohistochemistry

At day 20 post injury, the mice were anaesthetised and perfusion-fixed with 4 % paraformaldehyde in PBS. The spinal cord tissues, including injured regions, were immersed in 30% sucrose solution. Spinal cords were sagittally sectioned at a thickness of 12 μm using a CM3050S cryostat (Leica, Heidelberg, Germany). The sections were immunostained with rabbit anti-NF-H antibody (1:1000; Cat. No. AB1989, Chemicon, Temecula, CA, United States) and mouse anti-GFAP antibody (1:1000; Cat. No. G3893, Sigma-Aldrich) as primary antibodies. Alexa Fluor 594-conjugated goat anti-mouse IgG (1:400; Cat. No. A-11005, Life Technologies) and Alexa Fluor 488-conjugated goat anti-rabbit IgG (1:400; Cat. No. A-11008, Life Technologies) were used as secondary antibodies. Fluorescence images were obtained using an Axio Observer Z1 fluorescent microscope (Carl Zeiss, Oberkochen, Germany). Injured regions were defined by the GFAP-positive areas. The area of the injured region was measured using Image J analysis software (NIH, Rockville, MD, United States). Only fibre-like staining of NF-H was traced with Image J and the axonal density in each region was calculated. A blind observer analysed the images.

### Statistical Analysis

Statistical comparisons were performed by one-way analysis of variance (ANOVA) with the *post hoc* Bonferroni test, repeated measures two-way ANOVA with *post hoc* Bonferroni test, or an unpaired two-tailed *t*-test using GraphPad Prism 5 software (GraphPad Software, San Diego, CA, United States). *P* < 0.05 was considered statistically significant. The data are presented as the mean ± SE.

## Results

### NLK Treatment Promoted Secretion of Additional NLK From Astrocytes and Axonal Growth by Neurons

**FIGURE 1:**
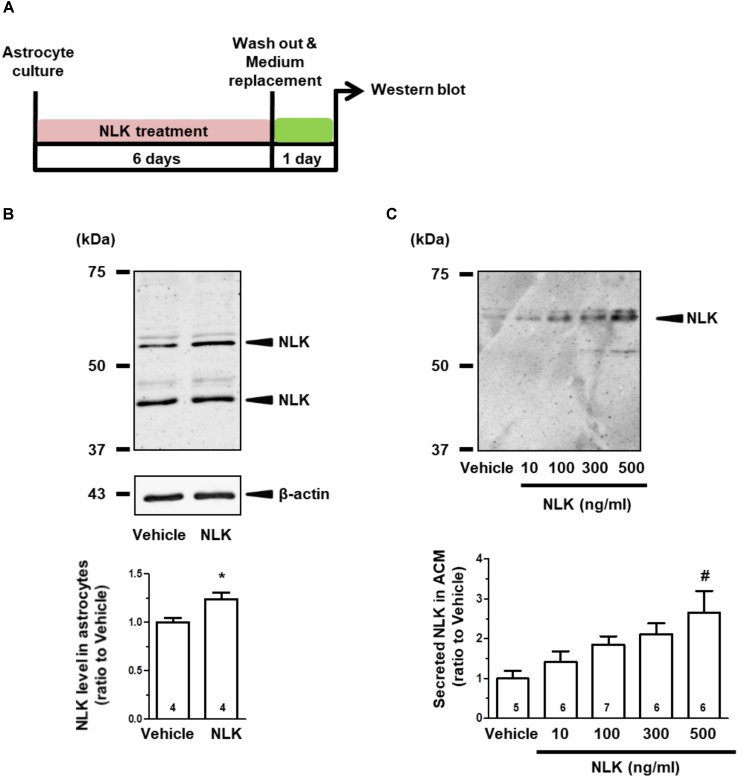
Neuroleukin (NLK) treatment enhances NLK secretion from cultured astrocytes. **(A)** Spinal cord astrocytes were cultured for 4 h and then treated with recombinant NLK (10, 100, 300, or 500 ng/ml) or vehicle for 6 days. The medium was replaced with supplement-free medium and 24 h later the medium was collected as astrocyte conditioned medium (ACM). **(B)** NLK in the lysates of cultured astrocytes was detected by western blotting. Band intensities of NLK in the lysates were quantified. ^∗^*p* < 0.05 vs. vehicle, unpaired two-tailed *t*-test. The numbers in the graph bars indicate the number of experiments. **(C)** NLK in ACM was detected by western blotting. Band intensities of NLK in ACM were analysed. ^#^*p* < 0.05 vs. vehicle, one-way ANOVA *post hoc* Bonferroni test. Numbers in the graph bars indicate the number of experiments.

Cultured astrocytes were treated with recombinant NLK for 6 days, subsequently washed to remove the recombinant NLK in the medium and on the cell surface, and then cultured for an additional 24 h to produce ACM (Figure 1A). Doublet bands were detected in the astrocytic lysate (Figure 1B). The bands detected were between 50 and 75 kDa in both the lysate (Figure 1B) and ACM (Figure 1C) and were in approximate agreement with molecular weight of AMF (a synonym of NLK) reported previously (Watanabe et al., 1996). The lower molecular weight protein in Figure 1B appeared to be a shorter variant of NLK, which is consistent with the intracellular cleavage form of AMF that is produced in human fibrosarcoma HT1080 cells (Niinaka et al., 1998). Both bands were considered in the current study to be NLK and their intensities were quantified. As a result of the NLK treatment, the expression level of NLK in the cell lysate was significantly elevated compared with that of the vehicle-treated group (Figure 1B).

The amount of NLK secretion from astrocytes was then measured. The level of NLK in ACM was increased by NLK treatment in a dose-dependent manner (Figure 1C). In addition, the molecular weight of the NLK in the ACM was slightly greater than that detected in the astrocytic lysate. The molecular weight of the NLK secreted from sertoli cells resembled that of the NLK in ACM (Deng et al., 2014), suggesting that the secreted NLK may have been influenced by some post-translational modifications. These results indicate that extracellular NLK enhanced the expression and secretion of NLK in astrocytes, suggesting an autocrine activity in the enhancement of NLK secretion in astrocytes.

**FIGURE 2:**
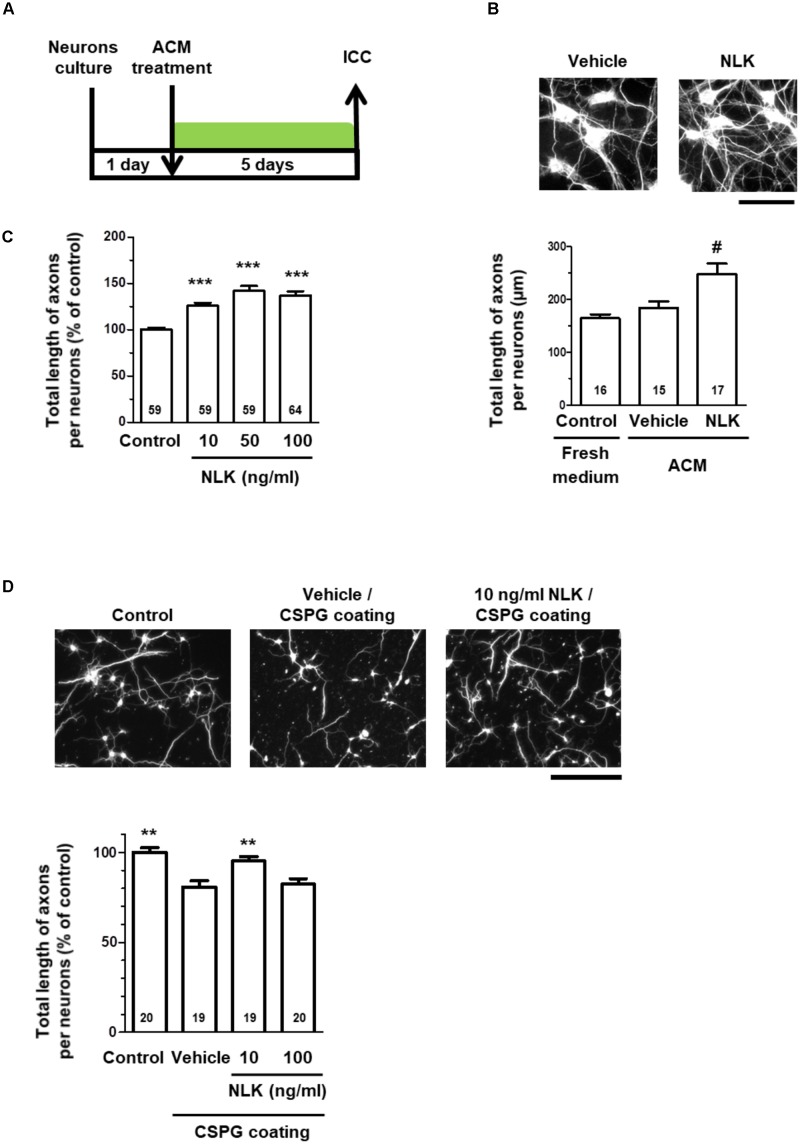
Neuroleukin (NLK) secreted from astrocytes may promote axonal growth in cultured neurons. **(A)** Cultured cortical neurons were treated with astrocyte conditioned medium *in vitro* at 1 day post culturing. Five days after the treatment, the cells were fixed and double immunostained for phosphorylated neurofilament-H (pNF-H) and microtubule-associated protein 2 (MAP2). **(B)** Representative images of pNF-H-positive axons are shown. Scale bar = 100 μm. Total lengths of pNF-H-positive axons per neuron were quantified for each treatment. ^#^*p* < 0.05 vs. vehicle, one-way ANOVA *post hoc* Bonferroni test. Numbers in columns indicate the number of captured images. **(C)** Cortical neurons were cultured *in vitro* for 1 day and then treated with recombinant NLK (10, 50, or 100 ng/ml) or vehicle (control) for 5 days. The cells were fixed and immunostained for pNF-H and MAP2. Total lengths of pNF-H-positive axons per neuron were quantified. ^∗∗∗^*p* < 0.001 vs. control, one-way ANOVA *post hoc* Bonferroni test. Numbers in the graph bars indicate the number of captured images. **(D)** Cortical neurons cultured in the presence of CSPG were treated with recombinant NLK (10 or 100 ng/ml) *in vitro* at 1 day post culturing. Four days after the treatment, the cells were fixed and immunostained for pNF-H and MAP2. Representative images of pNF-H-positive axons are shown. Scale bar = 100 μm. Total lengths of pNF-H-positive axons per neuron were quantified. ^∗∗^*p* < 0.01 vs. vehicle, one-way ANOVA *post hoc* Dunnett’s test. Numbers in the graph bars indicate the number of captured images.

Treatment of cultured neurons with ACM from astrocytes stimulated by NLK significantly increased the axonal density in the cultures (Figures 2A,B). This result suggests that astrocytes secreted some form of axonal growth factors following NLK stimulation. To investigate the axonal growth activity of NLK itself, cortical neurons *in vitro* at 1 day post culturing were treated with recombinant NLK or vehicle for 5 days. NLK treatment at concentrations of 10, 50, and 100 ng/ml significantly increased the axonal density compared with that of vehicle treatment (Figure 2C). In CNS traumatic injury, CSPGs are deposited, and inhibit axonal regeneration in the lesion site (Silver and Miller, 2004). To confirm whether NLK promotes axonal growth in the inhibitory environment, at 1 day *in vitro*, 10 or 100 ng/ml NLK or vehicle was added to cultured cortical neurons in CSPG-coated well. Four days post treatment, the axonal density was significantly increased by NLK stimulation at concentration of 10 ng/ml (Figure 2D). This result indicates that NLK has the axonal growth activity even in the presence of CSPG *in vitro*. From these results, NLK is expected to be one of the axonal growth factors secreted from astrocytes that was stimulated by the treatment with extracellular NLK. Our data suggest that an autocrine activity of NLK in astrocytes contributed to axonal growth in neurons via promoting NLK secretion.

### Autocrine Motility Factor Receptor Was Not Involved in the NLK-Induced NLK Secretion Signalling in Astrocytes

**FIGURE 3:**
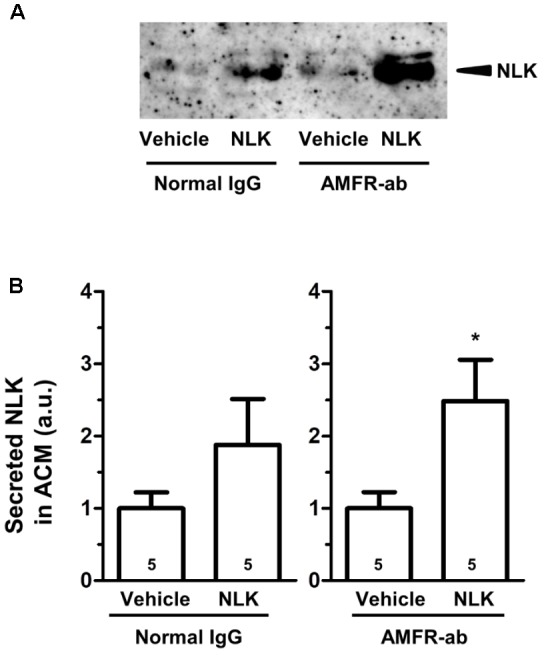
Autocrine motility factor receptor (AMFR) was not likely to be involved in the neuroleukin (NLK)-induced NLK secretion signal in cultured astrocytes. Spinal cord astrocytes were cultured for 4 days and then treated with AMFR neutralising antibody (AMFR-ab, 1 μg/ml) or normal IgG (1 μg/ml) for 15 min. Thereafter, NLK (500 ng/ml) was administered to the cells. At 5 days *in vitro*, the medium was replaced with serum-free medium, the cells were cultured for 24 h, and then the astrocyte conditioned medium (ACM) was collected. **(A)** NLK in ACM was detected by western blotting. **(B)** The band intensity of NLK in the ACM was quantified. ^∗^*p* < 0.05 vs. vehicle, unpaired two-tailed *t*-test. Numbers in the graph bars indicate the number of experiments.

As an NLK receptor, autocrine motility factor receptor (AMFR) is the only one reported (Fairbank et al., 2009). AMFR is a seven transmembrane-type receptor that promotes cell motility by NLK stimulation in tumour cells (Silletti et al., 1991; Shimizu et al., 1999). The expression of this receptor is also detectable in cultured neurons and astrocytes (Leclerc et al., 2000). A previous study suggested that NLK recognises an N-glycosylation site and C-terminal region of AMFR that are exposed to the extracellular space upon interaction with NLK (Haga et al., 2006). In the current study, we used a neutralising antibody to inhibit the binding of NLK to AMFR. This neutralising antibody was previously reported to inhibit AMFR by binding to C-terminal region of AMFR (Lucarelli et al., 2015). Primary cultured mouse spinal cord astrocytes were treated with NLK and the neutralising antibody for AMFR at the 4 th day in vitro culture. One day after treatment the amount of NLK in the ACM tended to increase as a result of NLK stimulation in both the IgG-treated groups and neutralising antibody-treated group (Figures 3A,B). This suggests that the blocking of AMFR did not influence NLK secretion that was induced by NLK treatment. Therefore, AMFR may not be involved in NLK-induced secretion of NLK from astrocytes.

### Extracellular NLK Interacted With GRP78

**FIGURE 4:**
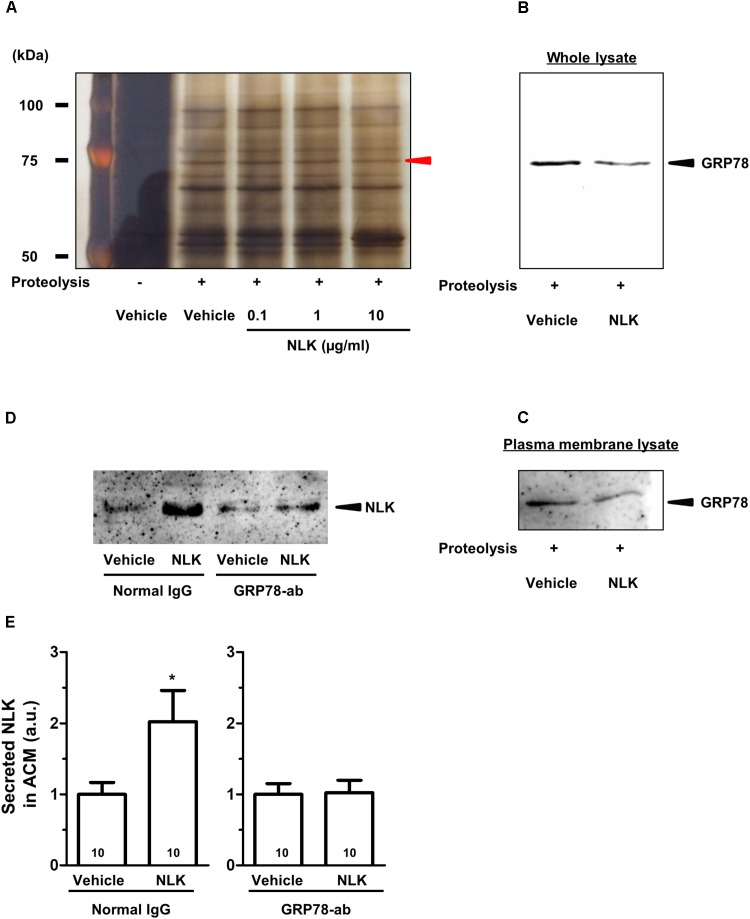
NLK bound to 78 kDa glucose-regulated protein (GRP78) in cultured astrocytes. **(A)** Following the drug affinity responsive target stability (DARTS) reaction, samples were electrophoresed and silver stained. The red arrowhead indicates a candidate band whose band thickness is different between the vehicle-treated lysates and NLK-treated lysates. The band was analysed using a Nano LC-MS/MS mass spectrophotometer and was identified as GRP78. **(B)** GRP78 after thermolysin digestion of cell lysates treated with vehicle or neuroleukin (NLK) and analysed by western blotting. **(C)** GRP78 after thermolysin digestion of membrane-associated lysates following treatment with vehicle or NLK and analysed by western blotting. **(D)** Spinal cord astrocytes were cultured for 4 days and then treated with a GRP78 neutralising antibody (2 μg/ml) or normal IgG (2 μg/ml) for 15 min. Thereafter, NLK (500 ng/ml) was administered to the cells. At 5 days post NLK-treatment *in vitro*, the medium was replaced with serum-free medium and the cells were cultured for 24 h to generate astrocyte conditioned medium (ACM). NLK in ACM was detected by western blotting. **(E)** The band intensity of NLK in ACM was quantified. ^∗^*p* < 0.05 vs. vehicle, unpaired two-tailed *t*-test. Numbers in the graph bars indicate the number of experiments independently performed.

To elucidate the mechanism of the NLK-induced NLK secretion, target proteins of extracellular NLK in astrocytes were explored using DARTS analysis (Lomenick et al., 2009). This method comprehensively identifies putative direct binding proteins of a ligand of interest. When the ligand binds to a protein, the structural conformation of the protein is modified. As a result, resistance against proteolysis is changed. Using this method, many ligand and receptor pairs have been identified (Qu et al., 2016; Yang et al., 2017; Tanabe et al., 2018). In the current study, cell lysate of astrocytes was incubated with recombinant NLK or vehicle. After proteolysis by thermolysin, a thinner protein band was detected using SDS–PAGE at approximately 75 kDa in the NLK-treated lysate compared with that in the vehicle-treated lysate (Figure 4A). Nano LC-MS/MS analysis indicated that the band was GRP78 (sequence coverage: 30%, score: 630). To confirm this result, western blotting for GRP78 was performed following thermolysin digestion. The amount of GRP78 in the thermolysin reactant pretreated with NLK was decreased compared with that in the vehicle solution-treated sample (Figure 4B). Hence, the structural change of GRP78 apparently occurred after binding with NLK, leading to accelerating proteolysis of GRP78. GRP78 belongs to the heat-shock protein 70 family and is a chaperone protein that facilitates the folding and assembly of its partner proteins, controlling the quality of the proteins and regulating endoplasmic reticulum stress signalling. Recent studies indicate a variety of localisation for GRP78, including the cell surface, cytoplasm, nucleus, endoplasmic reticulum, and extracellular space by secretion (Ni et al., 2011). GRP78 is also expressed at the plasma membrane in neurons and astrocytes (Goldenberg-Cohen et al., 2012). In the current study, after thermolysin digestion of the plasma membrane lysate prepared from cells treated with NLK, the amount of GRP78 decreased compared with that of lysate prepared from cells treated with vehicle (Figure 4C). This result showed the possibility that NLK bound to plasma membrane that was bound to GRP78. To investigate whether NLK increased NLK secretion via membrane-localised GRP78, the function of GRP78 was blocked by a neutralising antibody (Kelber et al., 2009). In normal IgG treated-groups, the amount of NLK in ACM was significantly increased by NLK stimulation. In contrast, the neutralising antibody treatment diminished NLK-induced NLK secretion from astrocytes (Figures 4D,E). These results suggested that cell-surface GRP78 played a critical role in NLK-induced NLK secretion in astrocytes.

### Injection of NLK to the Injured Site in SCI Increased Axonal Density and Improved Motor Dysfunction in Mice

**FIGURE 5:**
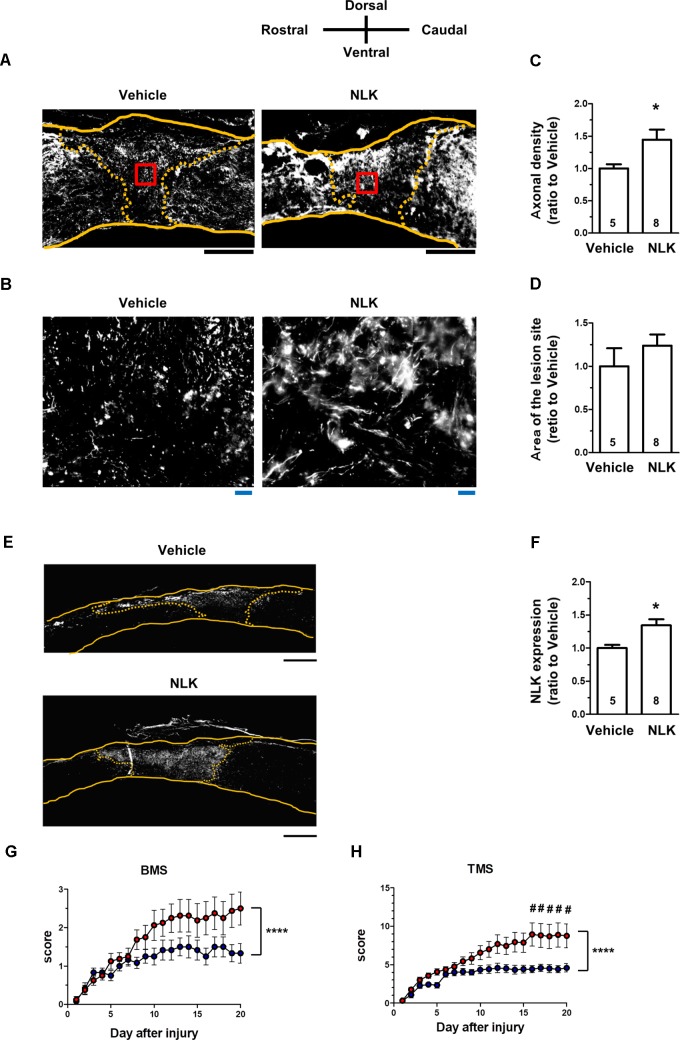
Injection of neuroleukin (NLK) into the injured site in spinal cord injury (SCI) mice induced axonal growth and improved motor function. SCI mice received microinjection of NLK (red circles, 8 mice, 16 hindlimbs, *n* = 16) or vehicle (blue circles, 6 mice, 12 hindlimbs, *n* = 12) within 15 min after injury. Twenty days after injury, spinal cords were collected and sagittal sections of the spinal cords were immunostained for phosphorylated neurofilament-H (pNF-H), glial fibrillary acidic protein (GFAP), and NLK. Representative immunofluorescence images of pNF-H **(A)** and NLK **(E)** are shown. Areas enclosed with red lines in **(A)** are magnified in **(B)**. The yellow lines show the outline of the spinal cords. The yellow dotted lines indicate the border of the glial scar region judged by immunostaining for GFAP. Density of pNF-H-positive axons **(C)**, size of the lesion site **(D)** and the expression level of NLK inside the lesion site **(F)** were quantified. ^∗^*p* < 0.05, vs. vehicle, unpaired two-tailed *t*-test. Black scale bars = 500 μm **(A,E)**, Blue scale bars = 20 μm **(B)**. Hindlimb motor function was evaluated using the Basso mouse scale [BMS **(G)**] and Toyama mouse score [TMS **(H)**]. ^∗∗∗∗^*p* < 0.0001 vs. Vehicle, drug × day interaction by repeated measures using two-way ANOVA. ^#^*p* < 0.05, vs. vehicle at each time point, *post hoc* Bonferroni test.

In SCI, motor dysfunction is caused by axonal disruption such as in the descending tracts. After injury, astrocytic glial scars surround the lesion sites and the astrocytes secrete CSPGs, resulting in inhibition of axonal growth and motor function (Silver and Miller, 2004). As shown in Figures 2C,D, extracellular NLK demonstrated axonal growth activity in cultured neurons. Then, effect of NLK on axonal growth was evaluated in SCI mice. In a previous report, the microinjection of recombinant acidic fibroblast growth factor (aFGF) to SCI rodents was performed (Tsai et al., 2008). In this report, 4 μg of recombinant aFGF was injected to the lesion site at the injured day, resulting in significant recovery of motor function. In addition, recombinant aFGF significantly increased neurite density on the order of ng/ml in cultured spiral ganglion neurons (Dazert et al., 1998). Therefore, the current study used the similar dose of recombinant NLK with aFGF. Recombinant NLK (2 μg/mouse) or vehicle was injected into the lesion site within 15 min after induction of the injury. At 20 days post injury, spinal cord tissues, including lesion sites, were isolated and sagittal sections prepared. These sections were immunostained for NF-H as an axonal maker (Figures 5A,B) and GFAP as a reactive astrocyte maker. The lesion site was defined as the region surrounded by GFAP-positive cells. There was no difference in the area size of the lesion sites between the NLK group and vehicle group (Figure 5D). The density of NF-H-positive axons at the lesion site was significantly increased in NLK-injected mice compared with that in the vehicle group (Figure 5C). This result suggested that the injection of NLK promoted axonal growth at the lesion site in SCI mice. In addition, the expression of NLK was significantly increased at the injured site of NLK-injected mice compared with that of vehicle-injected mice (Figures 5E,F). These results indicated that exogenous NLK promoted NLK secretion from astrocytes and contributed to the increase in axonal density at the lesion site.

The hindlimb motor functions were scored using the BMS (Basso et al., 2006; Figure 5G) and TMS (Shigyo et al., 2014; Figure 5H). BMS and TMS scores from 20 days of behavioural observation for NLK-injected mice were significantly elevated compared with those for vehicle-injected mice. In both sets of evaluation scoring, time × drug interactions were shown to be significantly different between the vehicle-treated group and NLK-treated group [*F*(20, 520) = 2.79, *p* < 0.0001 in BMS; *F*(20, 520) = 4.00, *p* < 0.0001 in TMS]. During the 16–20 days post injury, the *post hoc* Bonferroni test indicated that the TMS score of NLK-injected group was significantly higher than that of vehicle-injected mice at days 16 to 20.

## Discussion

It has been previously reported that astrocytes play beneficial roles by secreting axonal growth factors (Teshigawara et al., 2013; Shih et al., 2014). However, little is known about how astrocytes are activated to secrete the axonal growth factors. The current study demonstrated that NLK secretion from astrocytes was promoted by NLK stimulation and secreted NLK contributed to axonal growth. Furthermore, cell surface GRP78 in astrocytes was identified as a key molecule for secretion of NLK. NLK-GRP78 signalling is a novel finding as a mechanism for the secretion of the axonal growth factor NLK from astrocytes. The current study also is the first report to demonstrate that extracellular NLK had axonal growth activity and enhanced recovery from motor dysfunction in SCI mice. A single injection of NLK into the injured site induced a gradual recovery of motor function during the 20 days of observation (Figures 5G,H). After NLK injection, the level of NLK was significantly increased at the lesion site of SCI mice (Figures 5E,F). These results suggest that NLK secretion was induced by the NLK injection, which led to continuous amplification of NLK secretion from astrocytes and to axonal growth in the injured region.

Autocrine motility factor receptor is a receptor of NLK and is expressed in astrocytes (Leclerc et al., 2000). NLK has been reported to activate the MAPK pathway via AMFR, resulting in resistance to anticancer agents (Kho et al., 2013) and the production of matrix metalloproteinases in tumour cell lines (Haga et al., 2008). There have been no reports of NLK receptors other than AMFR. From the current results using a neutralising antibody for AMFR, AMFR was not likely associated to NLK-elicited NLK secretion in astrocytes. The present study suggested GRP78 is a novel receptor of NLK in astrocytes. While no reports have shown that extracellular NLK binds to GRP78 or regulates cellular functions by activating signalling in astrocytes, GRP78 is known to be a chaperone protein in the endoplasmic reticulum and belongs to heat-shock protein family. GRP78 also has a role as a cell surface receptor (Ni et al., 2011). Downstream signalling mediated by cell surface GRP78 has been reported in several studies. For instance, a complex of Cripto and GRP78 enhances tumour growth by inhibiting transforming growth factor-β signalling and by stimulating PI3K-Akt signalling and the MAPK pathway, which leads to pro-proliferative and pro-survival effects (Kelber et al., 2009). Until now, there have been no reports indicating the function of cell surface GRP78 in astrocytes. Further investigations are needed to clarify the downstream signalling of GRP78 in astrocytes, such as the PI3K-Akt and MAPK pathways.

Chondroitin sulphate proteoglycans are known to inhibit axonal regeneration at the lesion site after SCI (Silver and Miller, 2004). It has not been clarified whether NLK decreases CSPGs levels at the lesion site. We previously reported that several agents showed axonal growth activity without the alteration at the lesion site (Teshigawara et al., 2013; Tanabe et al., 2018). From our *in vitro* experiment (Figure 2D), NLK promoted axonal growth in the presence of CSPG. Therefore, NLK possibly induced axonal growth at the injured site even without CSPG reduction.

Although NLK is reported to demonstrate neuronal pro-survival effects and axonal growth activity *in vitro* (Gurney et al., 1986; Deng et al., 2014), the current study is the first to indicate the effect of NLK on axonal growth *in vivo*. While GRP78 is expressed in neurons (Goldenberg-Cohen et al., 2012), its involvement in the axonal growth activity of NLK has not been revealed. In our axonal growth assay *in vitro*, NLK treatment with concentrations of 10–100 ng/ml promoted axonal growth in the absence of CSPG (Figure 2C). However, in the presence of CSPG, the axonal density was increased by the treatment with 10 ng/ml NLK, but not by treatment with 100 ng/ml NLK (Figure 2D). From these results, there might be different mechanisms of NLK-induced axonal growth between presence and absence of CSPGs. Therefore, mechanisms of axonal growth of NLK may be not simple. We are currently using DARTS to attempt to identify the receptor for NLK in neurons. Elucidation of the mechanism of NLK in neurons will clarify novel signalling that will be able to be targeted to promote axonal growth.

Our study revealed the potential of GRP78 signalling in astrocytes to exert beneficial effects on axonal growth, suggesting that NLK-GRP78 signalling may be important for promoting the beneficial effects of astrocytes that secrete NLK. NLK itself and/or drugs targeting cell surface GRP78 may be developed as novel therapeutic agents for CNS traumatic injury.

## Author Contributions

YT, NT, TK, and CT designed the experiments and wrote the manuscript. YT conducted the experiments and analysed the data. CT supervised all the experiments and analysis.

## Conflict of Interest Statement

The authors declare that the research was conducted in the absence of any commercial or financial relationships that could be construed as a potential conflict of interest.

## Abbreviations

ACM: astrocyte conditioned medium
AMF: autocrine motility factor
AMFR: autocrine motility factor receptor
ANOVA: analysis of variance
BMS: Basso mouse scale
CSPGs: chondroitin sulphate proteoglycans
DAPI: 4′,6-diamidino-2-phenylindole
DARTS: drug affinity responsive target stability
DMEM: Dulbecco’s modified Eagle’s media
GFAP: glial fibrillary acidic protein
GRP78: 78 kDa glucose regulated protein
HRP: horse-radish peroxidase
MAP2: microtubule-associated protein 2
NLK: neuroleukin
PBS: phosphate-buffered saline
pNF-H: phosphorylated neurofilament-H
SCI: spinal cord injured
SDS–PAGE: sodium dodecyl sulphate-polyacrylamide gel
TMS: Toyama mouse score
T-TBS: tris buffered saline
